# Is Pain “All in your Mind”? Examining the General Public’s Views of Pain

**DOI:** 10.1007/s13164-021-00553-6

**Published:** 2021-05-04

**Authors:** Tim V. Salomons, Richard Harrison, Nat Hansen, James Stazicker, Astrid Grith Sorensen, Paula Thomas, Emma Borg

**Affiliations:** 1 Department of Psychology, Queen’s University, Kingston, ON Canada; 2 School of Psychology and Clinical Language Sciences, University of Reading, Reading, UK; 3 Department of Philosophy, University of Reading, Reading, UK; 4 Department of Philosophy, King’s College London, London, UK

## Abstract

By definition, pain is a sensory and emotional experience that is felt in a particular part of the body. The precise relationship between somatic events at the site where pain is experienced, and central processing giving rise to the mental experience of pain remains the subject of debate, but there is little disagreement in scholarly circles that both aspects of pain are critical to its experience. Recent experimental work, however, suggests a public view that is at odds with this conceptualisation. By demonstrating that the public does not necessarily endorse central tenets of the “mental” view of pain (subjectivity, privacy, and incorrigibility), experimental philosophers have argued that the public holds a more “body-centric” view than most clinicians and scholars. Such a discrepancy would have important implications for how the public interacts with pain science and clinical care. In response, we tested the hypothesis that the public is capable of a more “mind-centric” view of pain. Using a series of vignettes, we demonstrate that in situations which highlight mental aspects of pain the public can, and does, recognize pain as a mental phenomenon. We also demonstrate that the public view is subject to context effects, by showing that the public’s view is modified when situations emphasizing mental and somatic aspects of pain are presented together.

## Introduction

Pain has been a focal point for scientists and philosophers interested in how the body and mind interact to produce conscious experience. While the precise nature of the relationship between damage to a body part and the mental aspects of pain continues to be a subject of intense scholarly debate, there is widespread agreement amongst scientists and clinicians that pain has an essential mental component. This is reflected by the IASP’s definition of pain as “An unpleasant sensory and emotional experience associated with actual or potential tissue damage, or described in terms of such damage”, with the accompanying note concluding that pain is “always a psychological state” (IASP 1979).

However experimental philosophers have recently argued that this scholarly conceptualization of pain is at odds with the way the general public thinks about pain. Investigators examined whether participants endorsed (what the investigators take to be) the three components of a mental or what we will term “mind-centric” conceptualization of pain, namely privacy, subjectivity and incorrigibility (Sytsma 2010). In a direct challenge to the privacy component, Sytsma found that the public believed that two individuals can experience the same pain (e.g. they felt conjoined twins joined at the torso were feeling the same pain if a shared leg was injured), suggesting that the privacy of pain is a function of the fact that we tend not to share body parts or nervous systems, rather than a definitive aspect of pain (Sytsma 2010). Similarly Sytsma contested the subjectivity component by demonstrating the public’s belief that pains can be unfelt (e.g. when an individual is distracted from the pain of an injury) and, as such, are not dependent on the awareness of the perceiver (Sytsma 2010). Finally, Reuter and colleagues found that experimental participants believe that pain can be hallucinated (e.g. when the subject thinks that they are experiencing ankle pain but their state is an unexpected side effect of a new medication), challenging the incorrigibility component of the mind-centric conceptualization of pain (Reuter et al. 2014).

Evidence that the public conception of pain is “body-centric” (i.e rejection of the notions of privacy, subjectivity and incorrigibility and a view of pain as located in and exhausted by non-brain states of the body) is at odds with the latent conceptualisation underlying most basic and clinical science, and poses a challenge to public acceptance of chronic pain conditions, many of which are not clearly linked to readily apparent peripheral pathology. As such, it is critical to evaluate the experimental evidence underlying such claims. It has been argued that these findings demonstrate only that the public *can* view pain from a body-centric perspective, not that this is their exclusive view of pain (Borg et al. 2019a; Liu forthcoming). In this study, we test the hypotheses that findings suggesting the public conception of pain is body-centric are a function of the situations presented in those experiments, and that the public can, and does hold a more mind-centric view of pain than those results indicate.

## Methods

### Design

This study consisted of three separate experiments, run in separate samples. The first experiment sought to replicate Sytsma and colleagues’ findings in a new sample. The second used a different set of vignettes created to test the hypothesis that the general public is capable of viewing pain as a mental experience. In the third experiment we included vignettes from the first two experiments to examine whether the inclusion of vignettes priming a “mind-centric” interpretation would modify responses to vignettes priming a “body-centric” interpretation and vice versa. The methods for each experiment are described below. Following the presentation of the materials for each experiment, we have included a discussion of the philosophical justification for the design of those materials (i.e for the content of the vignettes), in the sections headed ‘*Philosophical Justification for the Design of the Vignettes”*.

### Participants

All participants were recruited via Amazon Mechanical Turk (MTurk). Samples of participants recruited through MTurk tend to be more representative of the general population in the United States than typical lab studies (Thomas and Clifford 2017). Studies were only made available to ‘Masters’, which are high performing workers on MTurk (with a HIT Approval Rate > 95%). As all studies hinged on the ability to read and fully understand the vignettes, we recruited from countries where English is the first language (Australia, Canada, Republic of Ireland, New Zealand, United Kingdom, and United States) and screened for English fluency.

Originally, 110 participants were recruited for each experiment. Participants who did not complete the experiment fully, or had empty datasets were excluded. For consistency, the same exclusion criteria were used across all three iterations of the study. A small proportion of datasets experienced technical errors while transferring the data to our server and demonstrated partial data recording and were excluded (Exp 1: *n* = 1; Exp 2: *n* = 3). Lastly, each participant was asked to record their impressions of the experiment on the final trial of the experiment. Participants who did not engage with this section, or whose feedback was unintelligible were also excluded due to lack of engagement (Exp 1: *n* = 2; Exp 2: *n* = 1; Exp 3: *n* = 0). After accounting for these factors, 96 participants completed experiment 1 (43 females; mean/sd age 41.0/12.0, range 26–74); 82 participants completed experiment 2; 108 participants completed experiment 3 (43 females; mean/sd age 34.4/10.2). Participants also reported education attainment for experiment 1 (college = 28, undergraduate = 52, postgraduate = 14 and PhD/MD = 1) and experiment 3 (high-school = 1, college = 30, undergraduate = 55 and postgraduate = 14). Due to a coding error, demographic data were not collected for experiment 2. We presume a similar demographic to the other experiments due to similar recruitment methodology.

### Ethical Considerations

The protocol was approved by University Research Ethics Committee at the University of Reading. Participants provided informed consent and received a small monetary reward, dependent on the number of vignettes within each experiment, on completion of the study (Experiment 1 = $1; Experiment 2 = $2; Experiment 3 = $1).

### Materials

In all studies, vignettes described a theoretical pain scenario. Participants were asked how much they agreed with a statement about the vignette on a scale of 1–7, where ‘1’ was “clearly agree” and 7 was “clearly disagree”. To avoid participants “opting out” of particular questions, we did not include “not sure” as an anchor at the mid-point of the scale, as had been done in previous vignette studies (Reuter et al. 2014; Sytsma 2010). To facilitate reading of the vignette and prevent participants from simply clicking through the study, a change of position and delay of 3 s was introduced before the Likert-scale appeared on each trial.

To facilitate comparison between questions, and allow directional interpretations of final data, all vignettes were re-coded prior to analysis such that low scores indicated a “body-centric” view and high scores indicated a more “mind-centric” view (i.e. ‘1’ represent the most body-centric view, ‘7’ represents the most mind-centric view).

### Experiment 1

Experiment 1 consisted of 4 vignettes taken from 3 different studies, all from Sytsma (2010), and the statements participants were asked to endorse are presented in Table 1.

**Table 1:** Vignettes and statements adapted from Sytsma and colleagues

Vignette	Statement
1. Tree in the Woods There is an old puzzle that many people are familiar with: “If a tree falls in the woods and no one is there to hear it, does it make a sound?” Philosophers have posed a similar question about pain: “If a person has badly injured her leg but isn’t paying attention to it, is there still a pain?” Some philosophers have argued that when you stub your toe, for example, the pain is not really located in the injured toe; rather, they hold that the pain is produced in your mind and is merely caused by the injured toe. Other philosophers have disagreed, arguing that the pain is really in the injured toe and is simply felt by the mind.	There is still pain in a badly injured leg even when the person is not aware of it.
2. Distracted Patient Doctors have observed that sometimes a patient who has been badly injured will get wrapped up in an interesting conversation, an intense movie, or a good book. Afterwards, the person will often report that during that period of time they hadn’t been aware of any pain.	In such a situation, the injured person still had pain and was just not feeling it during that period. There was no pain during that period.
3. Three-legged race Henry and Johnny are normal undergraduates at a state university. They are distinct people with their own beliefs and desires. One day they were participating in a three-legged race in a park with Henry’s right leg tied to Johnny’s left leg. While running toward the finish line their “third-leg” forcefully kicked a large rock that, unbeknownst to them, was hidden in the grass. Henry and Johnny both grimaced and shouted out “Ouch!”	They felt one and the same pain. They felt two different pains.
4. Conjoined twins (1): shared pain Bobby and Robby are conjoined twins that are joined at the torso. While they are distinct people, each with their own beliefs and desires, they share the lower half of their body. One day while running through a park they forcefully kicked a large rock that, unbeknownst to them, was hidden in the grass. Bobby and Robby both grimaced and shouted out “Ouch!”	They felt one and the same pain. They felt two different pains.

### Experiment *2*

For experiment 2, 8 novel vignettes were developed (Table 2). To ensure responses were not influenced by positive/negative phrasing, an alternate version was created in which statements were phrased in the negative. Versions were counterbalanced so that half of the participants got version A (positive) and the other half got version B (negative).

**Table 2:** Vignettes Used in Experiment 2

Vignette	Statement
1. Unexplained stomach pain Bill goes to hospital with all the observable symptoms of being in pain – for instance, he moans, grimaces, cries out, clutches his stomach and, if asked, says that he is in severe pain. However extensive investigations reveal that, since he started behaving in this way, Bill has suffered no tissue damage that could be responsible for his pain behaviour.	Bill is in pain
2. Brain stimulation Imagine that a surgeon discovers that stimulating a certain brain region in patients causes them to report feeling pain and to behave in other ways commonly associated with pain experiences (e.g. they cry out, grimace, say they are in pain, etc.). This stimulation causes no (non-brain) bodily changes.	The patient has pain
3. Wrist injury; No pain Maya is an ice skater who takes a bad fall on the ice and injures her wrist. At the time she says it doesn’t hurt, even though the damage to her hand is evident to all (it swells and bruises). Later on, looking back on the incident, Maya remarks “It was the darndest thing, although I couldn’t use my hand properly it never actually hurt, not when the accident happened, not when they were treating it at the hospital, and not when it was bandaged up. Although I could see the damage, it was never actually painful.”	Maya had pain from this injury
4. Congenital insensitivity There is a serious medical condition called congenital insensitivity to pain where patients report they do not feel pain. These patients have a very difficult life as they often injure themselves since they have no warning that injury is occurring (they pick up hot objects or cut themselves without realising it, for example). Patients with this condition often say that they are aware of some sensations in affected body parts (e.g. someone who breaks their leg may talk of a feeling of blood flowing to that limb) but they say they do not feel pain.	Patients have pain
5. Conjoined twins (2): unshared pain Adam and Zed are conjoined twins that are joined at the torso. While they are distinct people, each with their own beliefs and desires, they share the lower half of their body. One day while running through a park they forcefully kick a rock that, unbeknownst to them, was hidden in the grass. Adam grimaces and says ‘ouch’, Zed doesn’t. On examining their shared leg both agree that there is tissue damage (there is a scrape where it hit the stone) but while Adam says it hurts, Zed insists it doesn’t.	Adam has pain Zed has pain
6. Broken toe; no pain Jill has trained as a soldier and has, in many situations, suffered damage to her body which she has had to work through. Jill remembers breaking a toe when she was seven and it really hurting. Yet, during the course of an army exercise, Jill breaks the same toe again and this time she reports it doesn’t hurt.	Jill had pain when she broke her toe on the army exercise
7. Electric shock Murali is a scientist investigating whether memory can be enhanced by applying mild electric shocks to the skull to stimulate a particular brain region. However, he finds that whenever he applies mild shocks in this region, patients say that they suddenly start to feel a pain in their left hands after every shock. They also behave in other ways commonly associated with pain (e.g. they grimace and rub their left hand, they say that their hands hurt, etc.). The electrical stimulation causes no (non-brain) bodily changes.	The patients have pain in their left hand when the mild shock is delivered
8. Same stimulus, different pain Nisha is a scientist investigating pain. As part of this work she pushes a pin against the forearms of lots of different participants using the exact same pin and with the exact same force on each occasion. Nisha finds that people respond differently- some participants report this to be painful, some say that it is not painful.	All patients had pain

## Philosophical Justification for the Design of the Vignettes

These vignettes test the validity of different aspects of the mind-centric view. For instance, in the first vignette a potential parallel between pain and the experience of hearing a noise is drawn. As in the classic “if a tree falls in the forest…” question, answering that pain can occur without awareness indicates that pain is being viewed as an objective part of the world, not as something dependent on awareness. For vignette 1 (“Tree in the woods”), then, an answer which indicates participants think of pains as properties of body parts and as things which can exist whether or not the subject to which that body part belongs is aware of them, indicates a body-centric view. This challenge to the idea that ordinary people view pain as subjective (awareness-dependent) is also embodied in vignette 2 (“Distraction”), which explicitly probes the idea that pains can exist without being perceived.

Vignettes 3 and 4, on the other hand, focus on the alleged privacy of pain, i.e. the idea that a sensation of pain is only accessible to one individual. Together they press on the idea that, as Sytsma puts it, “it is the number of afflicted appendages, not the number of perceiving brains, that best corresponds with the number of pains reported” (Sytsma 2010). Vignette 3 (“Three-legged race”) is what we might label a standard case of pain attribution, where the number of afflicted appendages matches the number of perceiving brains (one afflicted appendage per person) and this leads subjects to attribute pains in a way that might seem to support judgements of privacy (judging that Henry has one pain and Johnny has another). The critical vignette in 4 (“Conjoined twins (1): shared pain”), however, presents a non-standard case – a set of conjoined twins sharing limb. Here the number of afflicted appendages (one) does not match the number of perceiving brains or people (two – Bobby and Robby). In this vignette, then, an answer which indicates that the situation involves only one pain (rather than two) suggests that claims about the privacy of pain are based on a mere typicality effect: Standard situations involve a matching number of afflicted appendages and perceiving brains. If we construct cases where the number of each no longer match, the appearance that people think of pains as private may simply evaporate, revealing an essentially body-centric view.

Finally, we note that in some of the vignettes we added additional statements to differentiate between having pain and awareness of having pain (this is discussed further below with respect to experiment 2). The body-centric view holds that these two states are dissociable (you can have a pain you are not aware of), whereas the mind-centric view holds that they are not (you cannot have pain without awareness). If we aim to examine which view the public is holding at any given time it is important to draw these two features apart but there is a risk that, without further contextualisation, a question such as ‘Did x have pain?’ will be heard by participants as simply equivalent to the question ‘Was x aware of having pain?’. To try to counteract this possibility, in certain vignettes we prefaced the relevant question (i.e. the question that actually probes whether the subject is holding a body-centric or a mind-centric view – which was always ‘did the subject *have/not have* pain?’) with a prior question about the subject’s *awareness* of pain (see discussion of experiment 2 below).

## Philosophical Justification for the Design of the Vignettes

Vignettes in experiment 2 were structured similarly to those in experiment 1, but each scenario draws apart the existence of bodily injury and the putative experience of pain. Vignettes were structured to do this as, if participants are willing or able to adopt a mind-centric view of pain, we would expect this perspective to be operative when judging scenarios where the experience of pain apparently exists independently from a bodily cause of pain (i.e. showing that participants allow that the existence of pain and the existence of non-brain bodily change can dissociate). Vignettes 1, 2 and 7 (“Unexplained stomach pain”, “Brain stimulation”, “Electric shock”) concern the possibility of pain which exists without non-brain-based bodily injury. It is worth noting here that, although probing the same kind of possibility, we opted for different prompts in these cases, with vignette 1 asking about whether the protagonist “is in pain”, while 2 and 7 ask whether the protagonist “has pain”.^1^

Vignettes 3 and 4 (“Wrist injury without reported pain”, “Congenital insensitivity”) concern the possibility that relevant bodily injury can occur without the existence of pain. Vignettes 5, 6 and 8 (“Conjoined twins (2): unshared pain”, “Broken toe; no pain”, “Same stimulus, different pain”) deal with the idea that one and the same bodily injury might constitute the existence of pain for one individual but not for another (even where a single afflicted appendage belongs to two different people), or for a single individual at one time but not at another.

5 of the 8 scenarios were followed by a single statement (e.g. ‘Bill is in pain’). Scenario 5 (“conjoined twins(s): unshared pain”) was followed by two prompts, to reflect the two potentially distinct perspectives (Adam’s and Zed’s) in play. Adam is an example of a subject who has relevant injury and who claims to be in pain, so we expected all participants to agree that Adam had pain in this situation, regardless of whether operating with a body- or mind-centric view. The experimentally relevant condition then concerned the comparison to answers about Zed: specifically, would participants who agreed that Adam had pain nevertheless still be willing to disagree with the claim that Zed had pain? Participants operating with a mind-centric view should be willing to allow this possibility; if pain is viewed as a property of our experience of (non-brain) bodily changes, then it should be possible for one and the same bodily change in one and the same shared limb to lead to the existence of pain for one person but fail to lead to the existence of pain for the other. On the other hand, participants operating with a body-centric view should not diverge in their answers in this way. If pain is a property of body parts and if it is agreed that Adam has pain (so that it is agreed that there is pain in the shared limb), this should be sufficient for guaranteeing the truth of the claim that “Zed has pain”.

Finally, scenarios 3 and 4 (“wrist injury; no pain”, “congenital insensitivity”) were also followed by two prompts – one asking about awareness of pain and one asking about having pain – but in these cases only the answer to the second question was experimentally relevant (thus only answers to the latter are included in the experimental analysis). As noted above, the two prompts were included in these cases in order to explicitly draw apart the idea that the subject is *aware* of a pain and the idea that the subject *has* a pain (which she may be unaware of). The vignettes presented made it clear that the subjects (either Maya or those suffering from congenital insensitivity to pain) are *not aware* of pain in the situations presented, thus we expected participants to agree with the claim that the subject in each vignette was not aware of pain, regardless of whether they were utilising a body-centric or a mind-centric viewpoint. Thus this question was not directly experimentally relevant (since answers given to it would not help to identify the view of pain held by the participant). However, we were concerned that providing a single prompt along the lines of:

- To what extent do you agree with the following statement: The subject in this situation had/did not have pain

risked participants tacitly answering a (probably more common) question about awareness of pain, rather than the key question about the having of pain *simpliciter.*

### Experiment 3

For experiment 3 we selected an equal number of body- and mind-centric vignettes (from experiment 1 and experiment 2, respectively). To match the valence of these questions as closely as possible, the three most body-centric questions (i.e. that elicited the highest body-centric response on the Likert scales), were selected and matched to the mind-centric vignettes whose average responses best matched these scores from a mind-centric-perspective. For example, on a 1–7 scale evaluating a “body-centric” vignette, the highest score of 7 (indicating strong agreement with a body-centric perspective) would be recoded to the lowest available response of 1 on a mind-centric scale. This recoding allowed us to present findings for body- versus mind-centricity on a single scale. It should be noted, however, that this is a presentational device, it does not entail a commitment to the conceptual claims that the body- versus mind-dimensions can be simply matched on a single ratio scale or that the two dimensions are necessarily opposed, with individuals always thinking of pain in one of these opposing ways; see the discussion of Murat Aydede’s act-object view in the Discussion section below.

### Analysis

#### Pre-Processing

During piloting, it was calculated that it took a minimum of 15 s to read and make a judgement of each vignette. As such, trials in which responses were < 15 s were judged invalid and removed.

To prevent participants from answering in an automatic fashion (e.g. simply clicking 7 for every response), some statements were reverse coded in the questionnaire, then reversed for analysis.

To evaluate replication of Sytsma’s original vignettes in Experiment 1, one-sample t-tests were run to test whether the responses were significantly higher than the mid-point of 4. We also tested the difference between results from Experiment 1 and the mean values reported by Sytsma using independent-sample t-tests between our dataset and the mean and standard deviation values reported in Sytsma’s original article (Sytsma 2010).

To test whether the novel vignettes within Experiment 2 elicited a mind-centric response, one-sample t-tests were run to test whether the responses were significantly higher than the mid-point of 4 on the Likert Scale.

To test whether responses to the body- and mind-centric sets of vignettes differed when presented in block format (i.e. homogenously mind-centric questions in isolation) as opposed to a mixed design (i.e. combination of mind- and body-centric vignettes within the same experiment), between-samples t-tests were run between questions from Experiments 1 and 2, and the identical questions within Experiment 3.

## Results

**Fig. 1:**
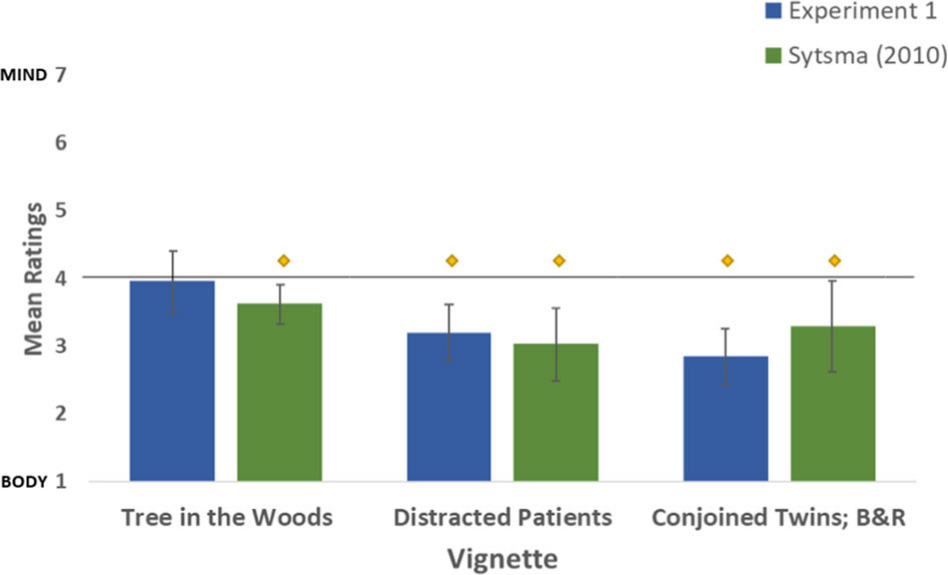
**Comparison of results from experiment 1 with mean scores reported by** **Sytsma (**2010**).** A low score on the y-axis represents a body-centric view of pain. Error bars represent 95% confidence interval. Diamonds represent significant difference from the mid-point of 4

Experiment 1 largely replicated findings by Sytsma et al. [9] (Fig. 1, Table 3), with most responses significantly less than 4 (Vignette 2, *M* = 3.21, *SD* = 2.09, *t*(62) = −3.98, *p* < 0.01; Vignette 3 *M* = 5.07, *SD* = 2.07, *t*(78) = 3.41, *p* < 0.01; Vignette 4: *M* = 3.04, *SD* = 2.08, *t*(57) = −5.31, *p* < 0.01, and not significantly different from Sytsma’s results (Vignette 2, t(135) = −0.50, *p* = 0.60; Vignette 3, t(129) = −0.87, *p* = 0.37; Vignette 4, t(129) = 0.6, *p* = 0.55).

**Table 3:** Experiment 1 results

Vignette	Mean	SD	Difference from Midpoint
1 “Tree in the Woods”	3.98	2.28	*t*(93) = −0.23, *p* = 0.82
2 “Distracted Patients”	3.21	2.09	t(62) = −3.98, *p* < 0.01
3 “Three Legged Race”	2.93	2.07	*t*(78) = 3.41, p < 0.01
4 “Conjoined Twins”	3.04	2.08	*t*(57) = −5.31, *p* < 0.01

The lone exception was vignette 1, which was not significantly less than 4 (*M* = 3.98, *SD* = 2.28, *t*(93) = −0.23, *p* = 0.82), although it was not significantly different from values obtained by Sytsma studies (vignette 1, t(333) = −1.3, *p* = 0.18).

**Fig. 2:**
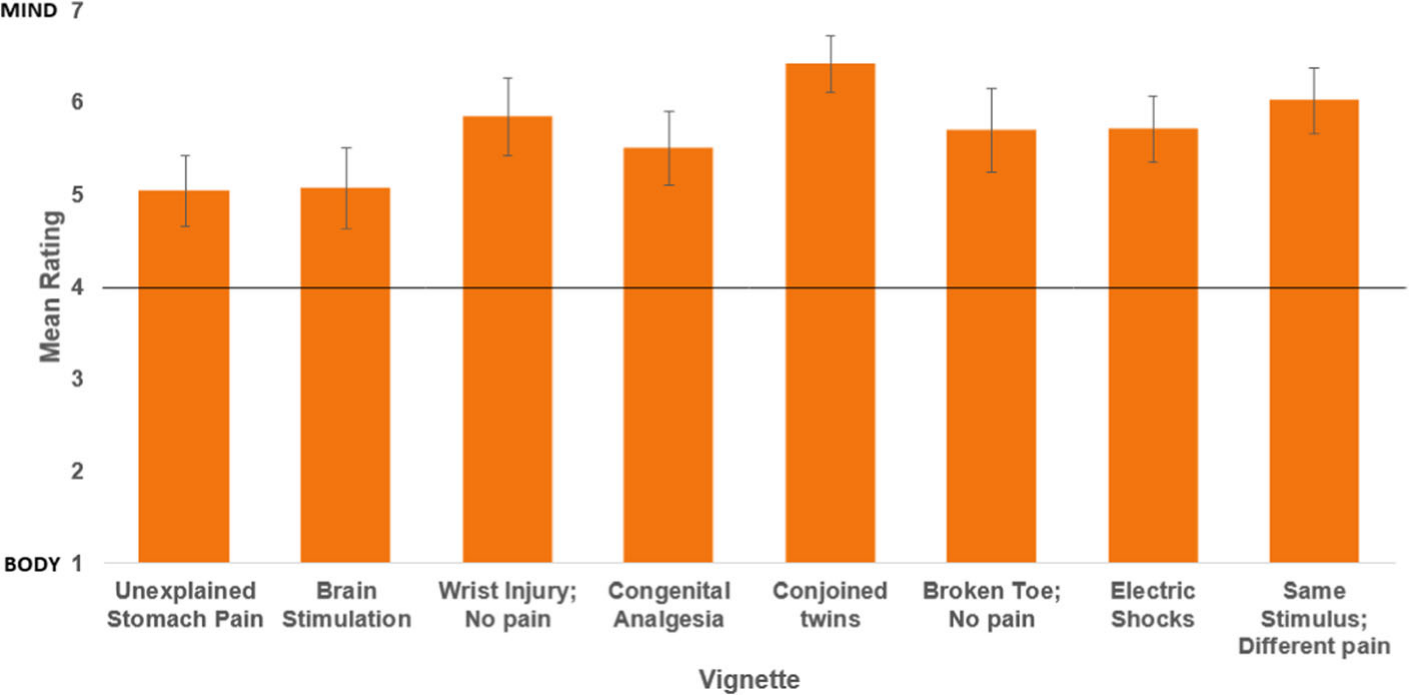
**Mean scores for Experiment 2**. A high score on the y-axis represents a mind-centric representation. Error bars represent 95% confidence interval. All vignettes displayed were significantly different to the mid-point of 4, demonstrating mind-centricity in participant responses

Consistent with our hypotheses, in experiment 2 all statements produced results that were significantly greater than 4 (Fig. 2, Table 4), which is evidence that participants endorse a mind-centric view of pain.

**Table 4:** Experiment 1 results

Vignette	Mean	SD	Difference from Midpoint
1 “Unexplained Stomach Pain”	5.04	1.8	*t*(79) = 5.17, *p* < 0.01
2 “Brain Stimulation”	5.07	2.08	*t*(74) = 4.45, p < 0.01
3 “Wrist Injury; No Pain”	5.84	1.95	*t*(74) = 8.16, p < 0.01
4 “Congenital Analgesia”	5.50	1.86	*t*(81) = 8.74, *p* < 0.01
5 “Conjoined Twins”	6.41	1.42	*t*(74) = 14.77, *p* < 0.01
6 “Broken Toe; No Pain”	5.69	2.11	*t*(75) = 9.05, *p* < 0.01
7 “Electric Shocks”	5.71	1.64	*t*(79) = 7.84, *p* < 0.01
8 “Same Stimulus; Different Pain”	6.01	1.65	*t*(73) = 10.49, *p* < 0.01

**Fig. 3:**
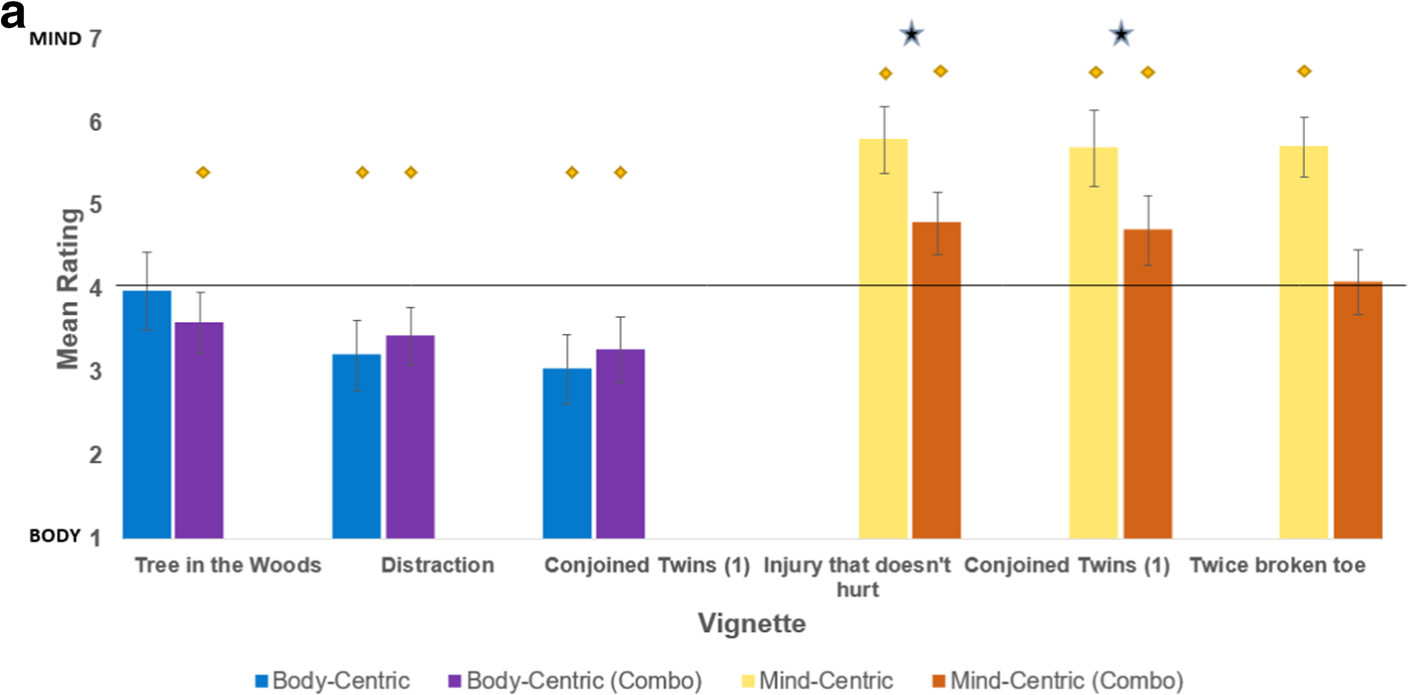

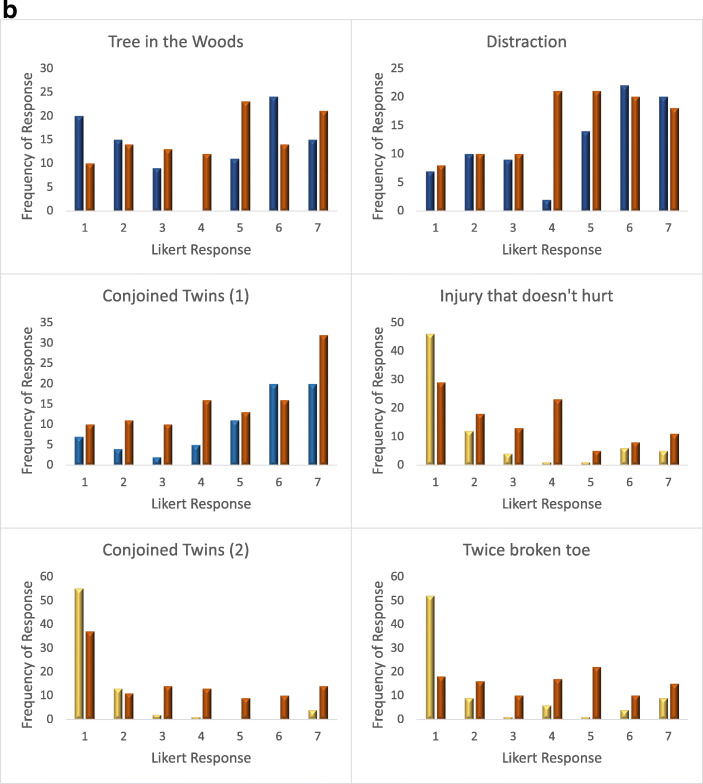
***A. means*** **of body-centric and mind-centric vignettes when presented in isolation, vs. in combination with each other (Combo).** Confidence interval bars displayed. Stars represents significant inter-experimental differences, diamonds represent significant difference from the mid-point of 4. **b. Frequency of responses to vignettes presented across Experiments 1, 2 and 3.** Responses to body-centric vignettes in isolation (Exp 1), are in blue. Responses to mind-centric vignettes in isolation (Exp 2) are in yellow. Responses to vignettes when combined together (Exp 3) are presented in orange

In experiment 3 we tested whether presenting mind-centric and body-centric vignettes together would result in different responses than when each was presented alone, in order to determine whether views of pain could be modulated by contextual information presenting a broader range of situations (Fig. 3a). There were no differences between responses on the three selected body-centric vignettes when they were presented alone in experiment 1 compared to when they were presented together with the mind-centric vignettes in experiment 3 (Vignette 1: t(202) = −0.91, *p* = 0.37; Vignette 2: t(202) = 1.36, *p* = .018; Vignette 3: t(202) = −0.71, *p* = .48). However, all three mind-centric vignettes elicited stronger effects (higher ratings) when presented in isolation, as opposed to when presented alongside body-centric vignettes (Vignette 4: t(190) = 5.96, *p* < 0.01; Vignette 5: t(190) = 13.19, p < 0.01; Vignette 6: t(189) = 3.18, p < 0.01) (Fig. 3b).

## Discussion

There has been increased focus in the medical and scholarly community on mental aspects of the pain experience and how to best understand and manage these aspects in a way that respects the privacy, subjectivity and incorrigibility of each individual’s pain experience. Recent work has suggested, however, that such a “mind-centric” view of pain might be at odds with the views of the general public, who see pain primarily as a feature of events occurring in the non-brain body, rather than a mental event that depends on consciousness. Thus, Reuter and Sytsma (2018: 1781) hold that the public “conceive of pains as being qualities of bodily disturbances—that is, as something that can be ‘merely instantiated,’ such that it might exist without being felt at a given time”.

If correct, the repercussions of this claim are significant and wide-ranging, for it suggests that the latent conceptualisation underlying most basic and clinical science is not concordant with that of the public. If, for example, it is the aim of a given intervention to ameliorate the suffering associated with pain (i.e. attend to the mental experience of pain, rather than focusing on disturbance in the somatic area where the pain is felt), a body-centric orientation on the part of the patient could be an impediment to acceptance of, and adherence to, the treatment plan. If the conceptualisation of pain underlying most clinical science is mind-centric (as captured in the IASP definition), and this conceptualisation is inconsistent with the public view, patients and clinicians may be destined to simply talk past one another when they try and communicate about pain. Finally, if it is true that the public holds a strong body-centric view of pain, it will have implications for public attitudes towards chronic pain conditions, many of which are not clearly linked to any apparent peripheral pathology.

We have previously hypothesized that although the evidence presented by these investigators demonstrates that the public *can* conceptualise pain from a body-centric perspective it doesn’t suffice to show that this is their exclusive view, for the public may also sometimes conceptualise pain from a mind-centric perspective (Borg et al. 2019b). Our results support this hypothesis, indicating that when presented with an alternative set of vignettes, participants from the general public can view pain as a mental experience, dependent on the individual’s consciousness and felt experience. As discussed below, although there remain divergent ways to accommodate these findings, our results support any view of our ordinary thought and talk about pain which allows variation, rather than the view that individuals possess a unitary body-centric way of approaching pain.

Our results also support the idea that an individual’s view of pain is malleable. We investigated whether mind-centric or body-centric views could be influenced by presenting vignettes together (that is to say, would someone be more or less likely to adopt a mind-centric view if prior context had demanded a body-centric view, and vice versa). We found that, although scores were closer to the mid-point for mind-centric views in experiment 3, the tendency to utilise a mind-centric view remained, even in a context where prior questions primed a body-centric view. Intriguingly, responses on body-centric items were not significantly different when the vignettes were presented together with mind-centric items, compared to when they were presented alone. A mundane but likely explanation for this finding is simple regression to the mean: responses on mind-centric questions were substantially further from the midpoint than body-centric items and, as such, were more likely to move towards the midpoint in mitigating conditions.

An alternative but untested explanation is that this malleability on mind-centric items reflects the fact that these items contain more ambiguity about the source of the pain. That is, they may be more susceptible to doubt when presented together with items that feature a more direct relationship between pain and a focal pathology. While we may, in our ordinary thought and talk about pain, be willing to allow that a target is in pain in the absence of a clear underlying somatic cause (as shown by experiment 2), this allowance may be undermined in a context where the intimate connection between pain and bodily injury has been brought to the fore (by presenting a body-centric vignette first). Such a hypothesis needs more careful testing but, if true, it would indicate that people are willing to view pain as a mental experience, but also to discount those mental experiences within the context of situations that suggest a more direct relationship between pain and somatic injury. We suggest that this could have significant repercussions for patient/clinician communication, which often takes place in a context where a more direct relationship between pain and somatic injury is salient.

When making a statement such as “I feel a pain in my left hand”, an individual is potentially sensitive to two distinct aspects of pain: that it is a felt state, akin to an emotion or other mental state, and that it is occurring in a body part. Reconciling these apparently mental and somatic aspects of pain has been an omnipresent theme of philosophical, scientific, and clinical examination of pain. Recent attention has turned to the views the public hold, and the extent to which these reflect thinking in academic and clinical circles. One approach to reconciling these distinct aspects of pain has been to view the folk notion of pain as inherently conflicted, as Hill (2005) does when discussing the “paradox of pain”. Aydede’s (2009) notion of “act-object duality” suggests that people sit relatively comfortably with both aspects of pain, not viewing their co-existence as paradoxical. The data presented here are consistent with either view, but suggest a certain situational malleability. Contextual elements apparently prompt some situations to be viewed through a body-centric lens, while others lead people to adapt a more mind-centric view.^2^

Finally, as noted above, the experimental results reported here are compatible with two alternative hypotheses about the source of variation in views of pain. On the one hand, the variation may (as has been suggested elsewhere, Borg et al. 2019a) be located at the level of thought, in different conceptualisations of pain. Alternatively, the variation may be located solely at the level of communicated content, for instance holding that our talk about pain varies between mental and bodily interpretations because the word “pain” is polysemous (Liu forthcoming). Further work will be needed to identify which of these competing hypotheses is correct and both remain live options.

We are currently investigating the potential existence of individual differences in views on pain and, if found, we think this would lend support to a conceptual account of variation. Examination of individual differences in pain-related cognitions have typically focused on an individual’s views of their own experienced pain, rather than on their views about pain as an abstract construct. An intriguing question raised by the findings reported in this paper is whether individuals hold stable views on pain and whether any such pre-existing views interact with how a subject experiences their own pain and/or responds to treatment for pain. While our results suggest that views of pain are, to some extent malleable (in the sense that they vary depending on the situation presented as well as the context in which the situation occurs), it remains to be seen whether such adjustments occur within a set range that is characteristic of each individual (Borg et al. forthcoming).

If stable individual differences can be found (i.e. if a given individual tends to provide more mind-centric answers to questions in a way that is stable across a range of prompts) we take it that this would indicate a more mind-centric conceptualisation of pain in that individual (mutatis mutandis for body-centric answers). A further question would then be whether any such difference in views influences an individual’s behaviour, both in terms of seeking treatment or support for their own pain, and responding to the pain of others. For instance, a patient who holds a body-centric view of pain might resist support in the absence of clear pathology, and an individual with a mind-centric view might be more likely to engage in treatments like cognitive behavioural therapy aimed at alleviating the mental burden of pain rather than any peripheral pathology. We suggest that these are important avenues to explore in further research on the clinical utility of the theoretical distinctions explored in this paper.

## Footnotes

1 In personal correspondence, Kevin Reuter helpfully queried this variability, suggesting that the phrase “is in pain” in fact promotes a mind-centric reading in a way that “has pain” does not. While this claim may be right, there are two points to note: first, our claim is that participants are capable of giving mind-centric responses depending on the ways in which pain situations are described to them, thus the idea that certain forms of words typically promote either a mind-centric or a body-centric reading, while an interesting suggestion and certainly worth further exploration, is not in opposition to the main claim of this paper. Second, as we will see with the experimental results below, the claim isn’t actually born out by our results, with vignette 7 (which asks about “has pain”) in fact eliciting more strongly mind-centric responses than either 1 or 2.

2 One element of the design of our experiments, inherited from the Sytsma and Reuter studies, is that body-centric and mind-centric judgments about pains are indicated on a single scale, which doesn’t allow for the possibility of the kind of co-existence judgments that Aydede predicts. Evaluating the possibility of the co-existence of body-centric and mind-centric judgments is a topic for further research.
